# Disclosure of domestic violence and sexual assault within the context of abortion: meta-ethnographic synthesis of qualitative studies protocol

**DOI:** 10.1186/s13643-017-0637-x

**Published:** 2017-12-15

**Authors:** Lydia Mainey, Annabel Taylor, Kathleen Baird, Catherine O’Mullan

**Affiliations:** 1 School of Nursing and Midwifery, CQUniversity Australia, Corner of Shields and Abbott Streets, Cairns, QLD 4078 Australia; 2 Queensland Centre for Domestic and Family Violence Research, CQUniversity Australia, Building C, City Campus, Mackay, QLD 4740 Australia; 3 School of Nursing and Midwifery, Menzies Health Institute Queensland, Griffith University, University Drive, Meadowbrook, QLD 4131 Australia; 4 School of Health, Medical and Applied Sciences, CQUniversity Australia, Bundaberg Campus, Bundaberg, QLD 4670 Australia

**Keywords:** Systematic review protocol, Domestic violence, Sexual assault, Health personnel, Abortion

## Abstract

**Background:**

One third of women will have an abortion in their lifetime (Kerr, QUT Law Rev 14:15, 2014; Aston and Bewley, Obstetrician & Gynaecologist 11:163–8, 2009). These women are more likely to have experienced domestic violence or sexual assault than women who continue with their pregnancies. Frontline health personnel involved in the care of women seeking abortions are uniquely positioned to support patients who choose to disclose their violence. Yet, the disclosure of domestic violence or sexual assault within the context of abortion is not well understood. To enhance service provision, it is important to understand the disclosure experience, that is, how frontline health personnel manage such disclosures and how victims/survivors perceive this experience. This review aims to provide a systematic synthesis of qualitative literature to increase understanding of the phenomena and identify research gaps.

**Methods:**

A meta-ethnography of qualitative evidence following PRISMA-P recommendations for reporting systematic reviews will be performed to better understand the experiences of domestic violence and sexual assault disclosure from the perspective of frontline health personnel providing support and women seeking an abortion. A three-stage search strategy including database searching, citation searching and Traditional Pearl Growing will be applied starting with the terms “domestic violence”, “sexual assault”, “disclosure” and “abortion”, their common synonyms and MeSH terms. The database search will include CINAHL, MEDLINE, Embase and PsycINFO. Published studies from 1970, written in English and from all countries will be included. Two reviewers will screen titles and abstracts and if suitable will then perform a full-text review. To attribute weight to each study, two reviewers will perform the critical appraisal using a modified version of the “Guidelines for Extracting Data and Quality Assessing Primary Studies in Educational Research”. Data extraction and coding will occur using EPPI-Reviewer 4 and will be carried out by two reviewers.

**Discussion:**

The reviewers will illuminate what transpires at the interface when women seeking an abortion in the context of domestic violence and sexual assault meet frontline health personnel. Increased knowledge in this area will improve the frontline health personnel’s practices and responsiveness to women who seek out healthcare in the context of violence.

**Systematic review registration:**

PROSPERO CRD42016051136.

**Electronic supplementary material:**

The online version of this article (10.1186/s13643-017-0637-x) contains supplementary material, which is available to authorized users.

## Background

One third of women in high-income countries elect to have an abortion in their lifetimes [1, 2]. Higher rates occur in emerging economies [3] where access to contraception is reduced and abortion is commonly unsafe and illegal. Women who have abortions experience domestic violence and sexual assault at up to three times the rate of those who continue with their pregnancies [2, 4–6]. This is because they are often subject to coercive and unprotected sex leading to a high rate of unplanned and unwanted pregnancies [2, 4, 7, 8].

Domestic violence and sexual assault are a major burden of disease in the global female population [9]. The United Nations identifies domestic violence and sexual assault as human rights violations, predominantly committed against women [10]. While many countries do not collect population-based data on domestic violence and sexual assault [5], Australian data suggests domestic violence and sexual assault cause the greatest health burden for women aged between 0 and 44 years of age [11].

Global research [4, 8] estimates that at least one third of women will experience domestic violence throughout their lifetime. Numerous studies have found that domestic violence escalates during pregnancy [9–12] and, consequently, many countries now advocate screening pregnant women for domestic violence over the course of their pregnancy and during their postnatal period [13, 14]. Unlike pregnant women who receive antenatal care and who have multiple opportunities to disclose domestic violence, women who undergo abortions are not routinely screened for domestic violence or sexual assault and possibly only come into contact with the health service once, at the time of their abortion, thereby reducing the opportunity for disclosure.

Frontline health personnel involved in the care of women seeking abortions have an important role to play in the enquiry and support for women experiencing domestic violence and sexual assault. Studies confirm that the act of discussing the experience of domestic violence with health personnel increases a woman’s likelihood of seeking assistance [12].

Quantitative systematic reviews exist in the field of domestic violence and abortion; the most recent review was conducted by Hall et al. in 2014 [6]. This review set out to determine an association between domestic violence and abortion. Before this, in 2009, Aston and Bewley conducted a scoping review of quantitative and qualitative studies to understand the relationship between domestic violence and abortion and to distinguish facts about domestic violence from cultural myths [2]. To our knowledge, there are no syntheses of qualitative studies on the disclosures of domestic violence and sexual assault in the context of abortion. As such, there is insufficient evidence to enhance service provision and support for women who seek healthcare in the context of domestic violence and sexual assault.

## Aims and objectives

The primary aim of this review is to synthesize qualitative research related to the disclosure of domestic violence and sexual assault within the context of women seeking an abortion. The objectives are to better understand disclosure and what has been shown to work well from (i) the woman’s perspective and (ii) the frontline health personnel’s perspective. This meta-ethnography will uncover how health personnel can be better supported to ask women about a history of domestic violence and sexual assault.

## Synthesis methods and methodology

This systematic review is registered with the International prospective register of systematic reviews (PROSPERO), registration number CRD42016051136. It will follow both the enhancing transparency in reporting the synthesis of qualitative research (ENTREQ) framework [13] and the Preferred Reporting Items for Systematic review and Meta-Analysis Protocols (PRISMA-P) [14]. PRISMA-P is included as Additional file 1 to this paper. A university librarian was consulted in designing of the protocol.

## Study design

In this review, we will use meta-ethnography to search, interpret and integrate the findings of qualitative research. Meta-ethnography, proposed by Noblit and Hare [15], is a systematic method for synthesizing qualitative research and has been widely used within the healthcare field to assimilate findings from individual patients and health professionals’ accounts [16]. It is particularly appropriate for questions related to experiences of care, has utility in synthesizing small numbers of studies and espouses to manage the different philosophical assumptions underpinning different types of qualitative research [15, 17].

This review will follow the seven-phase approach of meta-ethnography: (1) getting started, (2) deciding what is relevant to the initial interest, (3) reading the studies, (4) determining how the studies are related, (5) translating the studies into one another, (6) synthesizing translations and (7) expressing the synthesis [15].

*Phase 1: getting started*. The commencement phase occurred when LM identified the phenomenon of interest and the primary aim of the review was conceived and refined by the review team.

*Phase 2: deciding what is relevant*. Relevance will be discussed in the section below (“Approach to searching” section); the remaining five phases will be discussed in a later section (“Synthesis of findings” section). For clarity, each phase will be highlighted in italics throughout.

## Approach to searching

We will employ the SPIDER search strategy tool, developed by Cook et al. [18], to the review. The SPIDER tool (**S**ample, **P**henomenon of **I**nterest, **D**esign, **E**valuation, and **R**esearch Type) has been cited by the authors as being more suitable for generating search terms for qualitative research and returning a higher rate of relevant articles than PICO. Each aspect of the SPIDER tool will be discussed separately below:

### Sample

*Phase 2: deciding what is relevant to the initial interest*. The sample or population of interest is women seeking out abortions and the health personnel involved in this process. We will include qualitative studies which have reported data related to the experiences of women seeking an abortion and the experiences of their health personnel regarding domestic violence and sexual assault disclosures. While this scope is broad, an initial exploratory search revealed a manageable number of studies. All countries will be included in this study.

### Phenomenon of interest

We will include studies that document the screening or enquiry of domestic violence and sexual assault in women seeking an abortion in any healthcare context. Currently, “domestic violence” does not have a universal definition; therefore, for this review protocol, we will include studies which document any behaviour designed to coerce and control a current or ex-partner, child, stepchild, elder or another family member through intimidation and fear. This behaviour may include physical violence, sexual violence, emotional abuse, verbal abuse and intimidation, economic and social deprivation, damage of personal property and abuse of power [19].

Similarly, the definition of “sexual assault” is not ubiquitous and, for this protocol, will be understood as the forced or coerced sexual acts to a person, against their will and without consent [20]. Sexual assault may co-occur within the context of domestic violence (intimate partner sexual violence and incest) and outside of the family [21].

### Design

Studies conducted between 1970 and the present will be considered; 1970 marks the decade when domestic violence began to be understood internationally as a “high priority social issue” and not only a problem of the mentally ill or black women [22]. Studies that cite qualitative data collection methods, such as focus groups, interviews and narration, will be included.

### Evaluation

The term “research outcomes” is commonly used to describe the objective and observable results of quantitative studies. Qualitative research findings, however, may be subjective or unobservable [18]. Cook et al. [18] explain that the heading “evaluation”, rather than “outcome”, is more relevant to qualitative systematic literature review protocols. Studies eligible for inclusion in this literature review, therefore, will be those that have qualitative findings on the experiences of health personnel and women around domestic violence and sexual assault disclosures.

### Research type

Original qualitative studies, with detailed methodology and published in English, will be included as will commentaries or discussions on the subject, whereas books and grey literature will be excluded. We will consider qualitative studies to be research whose methods were intended to collect qualitative data and whose data were analysed qualitatively (e.g. phenomenologies, ethnographies, grounded theories and other coherent descriptions or explanations of phenomena). Mixed methods will also be considered if qualitative data are reported in full and can be extricated from other quantitative findings. Having ascertained an approach to searching, the next step is to conduct the literature search and selection of articles.

## Literature search and selection

### Electronic search strategy

An initial exploratory search using the terms “domestic violence”, “sexual assault”, “disclosure” and “abortion” was performed to obtain an overview of the topic and ensure an adequate number of citations were available to conduct the review. This approach has been used in other meta-ethnographies exploring complex topics [23]. Next, we will undertake the three-step search strategy outlined by Scott et al. [24] in their qualitative systematic review protocol. First, we will perform a scoping review of MEDLINE and CINAHL to identify keywords and phrases in titles and abstracts and MeSH/thesaurus terms used to index relevant articles. SPIDER headings and their associated search terms are presented in Table 1. A further search will then be undertaken across all identified databases by LM: CINAHL, MEDLINE, PsycINFO and Embase. Thesaurus terms will be translated and truncated as appropriate to each database. Where possible, spelling variations and lemmatization (recognition of different grammatical forms of a word) will be created as rules in the database searches. The list of citations will be screened for duplicates in the citation manager “Mendeley”; this has been shown to have a lower rate of false positive and false negative results than other citation managers [25]. The resulting catalogue of references will be referred to as the Start Set. A sample search strategy for MEDLINE is presented in Table 2. Finally, the electronic search will be supplemented by citation searching and Traditional Pearl Growing.

**Table 1:** Review headings and search terms

SPIDER heading	Search terms
Sample: healthcare providers OR females seeking abortion	unwanted pregnancy OR unplanned pregnancy OR abortion applicants OR induced abortion OR termination of pregnancy OR reproductive health OR reproductive health care OR RU486 OR Misoprostol OR Mifepristone AND patient care team or multidisciplinary care team OR health personnel OR nurses OR nurse OR nurse-patient relations OR medical staff OR doctor OR physician OR physician-patient relations OR professional-patient relations OR women OR female OR child OR adolescent OR young adult
Phenomenon of interest: domestic violence disclosure OR sexual assault disclosure	domestic violence OR child abuse OR spouse abuse OR violence OR intimate partner violence or gender-based violence OR battered women Sex offences OR rape OR sexual assault OR sexual violence OR incest OR victims OR date rape OR sexual coercion OR spousal rape OR forced sex AND disclosure OR self-disclosure OR truth disclosure OR communication OR enquiry OR conversation OR help seeking
Design: qualitative research	qualitative research OR interviews OR surveys and questionnaires OR grounded theory OR ethnography OR phenomenology OR focus groups OR content analysis OR thematic analysis OR constant comparative OR participant observation OR narrative OR filed notes
Evaluation: experience OR attitude OR practices	attitude of health personnel OR health knowledge, attitudes practice OR patient acceptance of health care OR perception OR practices OR intervention
Research type: qualitative or mixed methods	qualitative research OR qualitative analysis OR qualitative research OR mixed methods

**Table 2:** Sample MEDLINE search strategy

1. MeSH descriptor: [Pregnancy, Unwanted] this term only
2. MeSH descriptor: [Pregnancy, Unplanned] this term only
3. MeSH descriptor: [Abortion, Induced] explode all trees
4. MeSH descriptor: [Abortion Applicants] this term only
5. MeSH descriptor: [Abortion Habitual] this term only
6. MeSH descriptor: [Reproductive Health] explode all trees
7. MeSH descriptor: [Reproductive Health Services] explode all trees
8. Termination
9. Terminat* near/3 preg*
10. RU486 or mifepristone
11. Misoprostol
12. NOT Chromosome
13. NOT Abnormality
14. NOT Selective
15. (OR/ 1-14) AND (OR/ 12-14)
16. MeSH descriptor: [Patient Care Team] explode all trees
17. Multidisciplinary care team
18. Health* near/3 team
19. MeSH descriptor: [Patient Care Team] explode all trees
20. MeSH descriptor: [Health Personnel] explode all trees
21. MeSH descriptor: [Nurses] explode all trees
22. MeSH descriptor: [Physicians] explode all trees
23. MeSH descriptor: [Medical Staff] explode all trees
24. MeSH descriptor: [Nurse-Patient Relations] explode all trees
25. MeSH descriptor: [Physician-Patient relations] explode all trees
26. Medical staff
27. Doctor
28. MeSH descriptor: [Professional-Patient Relations] explode all trees
29. MeSH descriptor: [Women] explode all trees
30. MeSH descriptor: [Pregnant Women] explode all trees
31. Female
32. Child
33. MeSH descriptor: [Adolescent] explode all trees
34. MeSH descriptor: [Young Adult] explode all trees
35. OR/ 16-34
36. 15 AND 35
37. MeSH descriptor: [Battered Women] explode all trees
38. MeSH descriptor: [Intimate Partner Violence] this term only
39. MeSH descriptor: [Domestic Violence] this term only
40. MeSH descriptor: [Child Abuse] this term only
41. MeSH descriptor: [Spouse Abuse] this term only
42. Abuse near/3 women
43. Abuse near/3 partner
44. Abuse near/3 spous*
45. Wife near/3 batter*
46. Wife near/3 abuse
47. Violen* near/3 partner
48. Violen* near/3 dat*
49. MeSH descriptor: [Rape] this term only
50. MeSH descriptor: [Child Abuse, Sexual] this term only
51. MeSH descriptor: [Incest] this term only
52. Sex* near/3 abuse
53. Sex* near/3 coerc*
54. Sex* near/3 vioen*
55. Sex* near/3 assault
56. Rape near/3 dat*
57. Rape near/3 spous*
58. Force* near/3 sex OR force* near/3 intercourse
59. OR/37-58
60. MeSH descriptor: [Disclosure] this term only
61. MeSH descriptor: [Truth Disclosure] this term only
62. MeSH descriptor: [Self Disclosure] this term only
63. MeSH descriptor: [Communication] this term only
64. Enquir*
65. Convers*
66. Talk*
67. Information near/3 giving
68. Help near/3 seek*
69. Advice near/3 giving
70. OR/60-70
71. 59 AND 70
72. MeSH descriptor: [Qualitative Research] this term only
73. MeSH descriptor: [Focus Groups] this term only
74. MeSH descriptor: [Narration] this term only
75. MeSH descriptor: [Interviews as Topic] explode all trees
76. MeSH descriptor: [Surveys and Questionnaires] this term only
77. MeSH descriptor: [Grounded Theory] this term only
78. Phenomenology
79. Ethnography
80. Content Analysis
81. Thematic Analysis
82. Discourse Analysis
83. Constant Comparative Analysis
84. Participant Observation
85. Narrative
86. Field Notes
87. OR/72-86
88. MeSH descriptor: [Attitude] this term only
89. MeSH descriptor: [Attitude of Health Personnel] this term only
90. MeSH descriptor: [Knowledge] this term only
91. MeSH descriptor: [Comprehension] this term only
92. MeSH descriptor: [Intuition] this term only
93. MeSH descriptor: [Decision Making] explode all trees
94. MeSH descriptor: [Patient Acceptance of Health Care] explode all trees
95. MeSH descriptor: [Perception] this term only
96. MeSH descriptor: [Practices] this term only
97. MeSH descriptor: [Intervention] this term only
98. Experience
99. Impression
100. Concern
101. Understand*
102. OR/88-101
103. MeSH descriptor: [Qualitative Research] this term only
104. MeSH descriptor: [Qualitative Analysis] this term only
105. MeSH descriptor: [Mixed Methods] this term only
106. OR/103-105
107. 36 AND 71 AND 87 AND 102 AND 106

After the Start Set has been collected, title and abstract screening will be undertaken by LM and CO. Those articles which appear to meet the requirements of the review will directly undergo screening and appraisal processes which are detailed in a later section of this protocol. This is a crucial step as the reference lists of these studies will be used to identify addition relevant studies for the review. If this step is omitted, and further citations are included based on a study that is later excluded in the screening and appraisal phases, then a “rollback” must occur. In this context, a rollback refers to the removal of all additional citations identified from the reference list of the article that is excluded [26].

After screening and appraisal have occurred, Wohlin’s approach to Start Set citation searching will be used [26]. In the first iteration, the reference lists of the Start Set will be searched by LM and CO. Studies identified as potential candidates will be initially included or excluded based on the basic study screening criteria. Studies already in the Start Set will be excluded. Next, studies identified as potential candidates will be sorted through the screening and appraisal process to prevent rollback. Further iterations of reference list searching will continue until no new papers are found.

After this process, a search for papers citing the Start Set will be conducted in Web of Science which has been shown to be more reliable and cost effective than Google Scholar [27]. Studies identified as potential candidates will be initially included or excluded based on the basic study screening criteria. Studies which have already been found in previous citation searches will be excluded. Once this has occurred, the remaining papers will be filtered through the screening and appraisal processes. Further iterations of citation searching will continue until no new papers are found. Each iteration of this entire process will be completed separately to maintain repeatability and traceability [26].

**Fig. 1:**
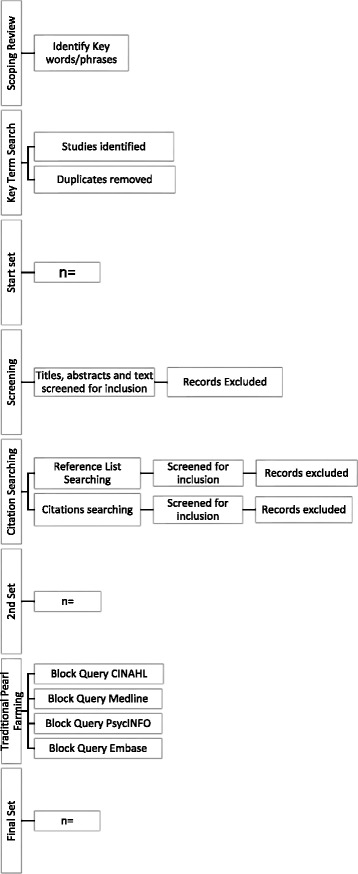
Search strategy

The resulting set of relevant articles will then be used for “Traditional Pearl Growing” as set out by Schlosser et al. [28]. The terms under which individual articles are indexed in CINAHL will be reviewed. Further relevant articles in CINAHL will be sought using the same index terms in a Building Block query. These steps will be repeated for MEDLINE, PsycINFO and Embase and will be undertaken by LM with guidance from CO. The studies identified through Traditional Pearl Growing will be filtered through the screening and appraisal processes (Fig. 1).

Throughout the literature search and selection process, a two-stage screening process and critical appraisal will be undertaken as detailed below.

### Study screening

#### Stage 1

LM and CO will individually screen study titles and abstracts to ensure they meet the inclusion criteria for the review. Upon completion of the screening process, the reviewers will meet to discuss which studies should be included or excluded from the review. If reviewers disagree, they will discuss until they arrive at a consensus. If consensus cannot be reached, LM will make a judgement call regarding the inclusion or exclusion of the study. Should the reviewers unanimously consider the study is unclear, LM will attempt to contact the study author for clarification. The author will have 2 weeks to reply before the article is excluded on the basis of insufficient information.

#### Stage 2

New studies, identified through citation searching and Traditional Pearl Growing, will be reviewed by LM and CO to ensure they meet the inclusion criteria as outlined above. This will occur with each iteration of backwards and forwards snowballing to mitigate a rollback of studies.

## Appraisal

The quality of studies which pass the screening process will be assessed according to the Review Guidelines for Extracting Data and Quality Assessing Primary Studies in Educational Research [29]. These guidelines have been modified and tested by LM and KB to ensure they apply to the healthcare context (refer to Additional file 2). LM and CO will individually appraise each study based on the quality of its aims and rationale, research question and practice focus, sampling strategy, recruitment and consent, data collection and analysis, results and conclusions [29].

Studies will be assigned two types of “weight of evidence” scores as described in Bonell et al. [30] and Lewin et al. [31]. Reviewers will first assign a weight (low, medium, high) to rate the reliability of the findings and secondly to rate of usefulness/relevance of the findings (relevant, indirect relevance, partial relevance, uncertain relevance) [31]. LM and CO will meet to discuss their appraisals, and if the reviewers disagree on the weight of an individual study, they will converse until they arrive at a consensus. In the unlikely event that the two reviewers disagree, LM will make a judgement call regarding the weight of evidence ascribed to the study.

The assessment and explanation of these assessments will be presented in the review appendices, and review authors will describe the concerns regarding the extent to which the review findings reflect the phenomena of interest [31].

## Synthesis of findings

Meta-ethnography is a “comparative approach to the synthesis of published research studies” [32]. A key component of this approach is to identify the key concepts [16] or emerging themes [17] of each study and translate these into the themes of another study. Themes are used by social scientists to unify different epistemological approaches [33] in the same way that a meta-analysis is used by positivists to bring together multiple studies [15]. By using three different methods of synthesis, namely “reciprocal translation” (identifying overarching concepts), “refutational synthesis” (exploring contradictions between studies) and “line of argument synthesis” (building up a picture of the whole), meta-ethnography moves beyond a traditional narrative literature review to generate higher order theories about experiences [17].

### Data extraction

*Phase 3: reading the studies*. Individual studies will be entered into systematic review software EPPI-Reviewer 4 by LM. Each study will be read and reread individually by LM and CO until key themes emerge. These will be coded in EPPI-Reviewer 4, and memos will be used to create a key list of phrases, ideas and concepts [15]. Guidance and support will be given by AT and KB.

During this phase, LM and CO will also assess the “adequacy of data” [31]. This will be judged by assessing if saturation of key ideas or concepts has occurred and by considering the extent to which additional data are likely to change the findings. This will be noted and reported in the review.

*Phase 4: determining how the studies are related*. In this phase, LM and CO will compare individual studies along with the key themes, lists of phrases, with other studies to make an initial tentative assumption about the relationship between them [15]. The relationship between studies will determine the specific synthesis to be employed.

Studies that appear to be directly comparable will undergo reciprocal translation*.* This approach translates the research findings into each other to generate overarching themes [32]. On the other hand, if the combined studies appear to have findings which are in opposition to each other, refutational translation synthesis will occur which will explain the contradictions and differences. Studies that do not appear to be directly comparable, but at the same time are not in opposition, will undergo line-of-argument translation synthesis [15]. Line-of-argument synthesis “develops a picture of the whole phenomenon under study from the studies of its parts” [32].

*Phase 5: translating the studies into one another*. It is possible that the initial tentative relationship drawn between studies will be incorrect. Should this happen, the review will lack coherence. Incoherence can occur when the main finding is challenged by contrasting findings [31]. Lack of coherence will become apparent during phase five. In this phase, an analogy will be selected by LM and CO which encapsulates the themes, key findings and concepts of each and how they relate to one another. The translation must protect the individual themes. This allows themes to be compared between studies. If analogies cannot easily be found, then all reviewers will re-evaluate the relationship between studies as the individual themes, and their comparisons with each other, will be the likely cause of confusion [15].

*Phase 6: synthesizing translations*. In this phase, LM and CO will bring the translations and themes together and create them into something greater than their parts [15]. Translations of themes will be compared and analysed to determine if one can encompass those of other translations. This is considered a second-level synthesis where competing translations are translated into each other [15].

*Phase 7: expressing the synthesis*. The meta-ethnography is intended for the health policy audience. This audience has a specific language for which AT is intimately aware. The purpose of expressing this synthesis in the language of the health legislator is to allow them to see the phenomena from the frontline health personnel’s perspectives. AT will provide LM with guidance in the write-up phase of this study for publication.

## Limitations and strengths

In an attempt to enhance the consistency and transparency of this review, a structured approach to examining and appraising the limitations and strengths of the findings will be applied. Using the approach to assess confidence in findings put forward by Lewin et al., four components of confidence, namely methodological limitations, relevance, coherence and adequacy of data, will be assessed. Methodological limitations and relevance will be assessed by LM using the modified Review Guidelines for Extracting Data and Quality Assessing Primary Studies in Educational Research [29]. The assessment and explanation of these assessments will be presented in the review appendices [31]. Coherence will be assessed in the synthesis. An analogy which encompasses all findings of the review must be found. If not, then the relationship between studies has been misinterpreted and will be reviewed [15]. Adequacy of data refers to the richness and quantity of data. While traditional systematic reviews attempt to identify every study conducted on a particular topic, meta-ethnography uses theoretical sampling until data saturation is achieved. One criticism of this approach is that it is unclear how saturation is achieved when there is limited access to first-order constructs [17]. LM and CO will determine thematic saturation by considering the degree to which additional research findings are likely to change the findings of the review. An inadequate set of data may arise where (i) qualitative researchers have used descriptive titles which have been inappropriately indexed [17], (ii) the review question was too narrow or (iii) more primary research needs to be conducted in the substantive area [31]. The adequacy of data will be noted in the review.

## Discussion

This systematic review will present a synthesis of qualitative research related to the disclosures of domestic violence and sexual assault in the context of abortion. The review will help to uncover how health personnel support their clients. The review may also uncover how health personnel can be supported to ask women about a history of domestic violence and sexual assault. Finally, conducting this review will gather information on the types of studies already conducted on this topic, which will prevent the duplication of research.

## Additional files

Additional file 1: PRISMA-P (Preferred Reporting Items for Systematic review and Meta-Analysis Protocols) 2015 checklist: recommended items to address in a systematic review protocol. (DOC 77 kb)

Additional file 2: Modified review guidelines for extracting data and quality assessing primary studies in educational research. (DOC 240 kb)

## Abbreviations

ENTREQ: Enhancing transparency in reporting the synthesis of qualitative research
PROSPERO: International prospective register of systematic reviews
