# Supplementary material for: Disclosure of domestic violence and sexual assault within the context of abortion: meta-ethnographic synthesis of qualitative studies protocol
Source: Syst Rev. 2017 Dec 15;6:257. doi: 10.1186/s13643-017-0637-x (PMC5732414; doi:10.1186/s13643-017-0637-x)
Supplement: Supplementary file 1 — PRISMA-P (Preferred Reporting Items for Systematic review and Meta-Analysis Protocols) 2015 checklist: recommended items to address in a systematic review protocol. (DOC 77 kb) [file 13643_2017_637_MOESM1_ESM.doc]

**PRISMA-P (Preferred Reporting Items for Systematic review and Meta-Analysis Protocols) 2015 checklist: recommended items to address in a systematic review protocol***

| Section and topic | Item No | Checklist item |
| --- | --- | --- |
| ADMINISTRATIVE INFORMATION | | |
| Title: |  |  |
| Identification | 1a | Identify the report as a protocol of a meta-ethnographic synthesis of qualitative studies |
| Update | 1b | n/a |
| Registration | 2 | CRD42016051136 |
| Authors: |  |  |
| Contact | 3a | Page 1 |
| Contributions | 3b | Page 15 |
| Amendments | 4 | n/a |
| Support: |  |  |
| Sources | 5a | Page 15 |
| Sponsor | 5b | Nil |
| Role of sponsor or funder | 5c | N/A |
| INTRODUCTION | | |
| Rationale | 6 | Page 4-5 |
| Objectives | 7 | Page 6-7 |
| METHODS | | |
| Eligibility criteria | 8 | Page 6-8 |
| Information sources | 9 | Page 8 |
| Search strategy | 10 | Table 1 |
| Study records: |  |  |
| Data management | 11a | Page 11 |
| Selection process | 11b | Page 9 & 10 |
| Data collection process | 11c | 11-13 |
| Data items | 12 | 11-12 |
| Outcomes and prioritization | 13 | Page 8 |
| Risk of bias in individual studies | 14 | Page 10 & 14 |
| Data synthesis | 15a | Page 12 & 13 |
|  | 15b | n/a |
|  | 15c | n/a |
|  | 15d | n/a |
| Meta-bias(es) | 16 | n/a |
| Confidence in cumulative evidence | 17 | Describe how the strength of the body of evidence will be assessed (such as GRADE) |

*** It is strongly recommended that this checklist be read in conjunction with the PRISMA-P Explanation and Elaboration (cite when available) for important clarification on the items. Amendments to a review protocol should be tracked and dated. The copyright for PRISMA-P (including checklist) is held by the PRISMA-P Group and is distributed under a Creative Commons Attribution Licence 4.0.**

*From: Shamseer L, Moher D, Clarke M, Ghersi D, Liberati A, Petticrew M, Shekelle P, Stewart L, PRISMA-P Group. Preferred reporting items for systematic review and meta-analysis protocols (PRISMA-P) 2015: elaboration and explanation. BMJ. 2015 Jan 2;349(jan02 1):g7647.*
