# Supplementary material for: Disclosure of domestic violence and sexual assault within the context of abortion: meta-ethnographic synthesis of qualitative studies protocol
Source: Syst Rev. 2017 Dec 15;6:257. doi: 10.1186/s13643-017-0637-x (PMC5732414; doi:10.1186/s13643-017-0637-x)
Supplement: Supplementary file 2 — Modified review guidelines for extracting data and quality assessing primary studies in educational research. (DOC 240 kb) [file 13643_2017_637_MOESM2_ESM.doc]

**Guidelines for extracting data and quality assessing primary studies in educational research Version 0.9.7**

| **Section A: Administrative details** *Use of these guidelines should be cited as: EPPI-Centre (2003) Review Guidelines for Extracting Data and Quality Assessing Primary Studies in Educational Research. Version 0.9.7. London: EPPI-Centre, Social Science Research Unit.*   | A.1 Name of the reviewer | | A.1.1 Details | | --- | | | --- | --- | --- | | A.2 Date of the review | | A.2.1 Details | | --- | | | A.3 Please enter the details of each paper which reports on this item/study and which is used to complete this data extraction. *(1): A paper can be a journal article, a book, or chapter in a book, or an unpublished report.  (2): This section can be filled in using bibliographic citation information and keywords 1, 2, and 4 from the EPPI-Centre Core Keywording Strategy (V0.95)* | | A.3.1 Paper (1)  *Fill in a separate entry for further papers as required.* | | --- | | A.3.2 Unique Identifier: | | A.3.3 Authors: | | A.3.4 Title: | | A.3.5 Source: | | A.3.6 Status: | | A.3.7 Language: | | A.3.8 Identification of report: | | A.3.9 Paper (2) | | A.3.10 Unique Identifier: | | A.3.11 Authors: | | A.3.12 Title: | | A.3.13 Source: | | A.3.14 Status: | | A.3.15 Language: | | A.3.16 Identification of report: | | | A.4 Main paper. Please classify one of the above papers as the 'main' report of the study and enter its unique identifier here. *NB(1): When only one paper reports on the study, this will be the 'main' report.  NB(2): In some cases the 'main' paper will be the one which provides the fullest or the latest report of the study. In other cases the decision about which is the 'main' report will have to be made on an arbitrary basis.* | | A.4.1 Unique Identifier: | | --- | | | A.5 Please enter the details of each paper which reports on this study but is NOT being used to complete this data extraction. *NB (1): A paper can be a journal article, a book, or chapter in a book, or an unpublished report.  NB (2): This section can be filled in using bibliographic citation information and keywords 1, 2, and 4 from the EPPI-Centre Core Keywording Strategy (V0.95).* | | A.5.1 Paper (1) *Fill in a separate entry for further papers as required.* | | --- | | A.5.2 Unique Identifier: | | A.5.3 Authors: | | A.5.4 Title: | | A.5.5 Source: | | A.5.6 Status: | | A.5.7 Language: | | A.5.8 Identification of report: | | A.5.9 Paper (2) | | A.5.10 Unique Identifier: | | A.5.11 Authors: | | A.5.12 Title: | | A.5.13 Source: | | A.5.14 Status: | | A.5.15 Language | | A.5.16 Identification of report: | | | A.6 If the study has a broad focus and this data extraction focuses on just one component of the study, please specify this here. | | A.6.1 Not applicable (whole study is focus of data extraction) | | --- | | A.6.2 Specific focus of this data extraction (please specify) | | |
| --- | --- | --- | --- | --- | --- | --- | --- | --- | --- | --- | --- | --- | --- | --- | --- | --- | --- | --- | --- | --- | --- | --- | --- | --- | --- | --- | --- | --- | --- | --- | --- | --- | --- | --- | --- | --- | --- | --- | --- | --- | --- | --- | --- | --- | --- | --- | --- | --- | --- |
| **Section B: Study aim(s)and rationale**   | B.1 What are the broad aims of the study? *Please write in authors’ description if there is one. Elaborate if necessary, but indicate which aspects are reviewers’ interpretation. Other, more specific questions about the research questions and hypotheses are asked later.* | | B.1.1 Explicitly stated (please specify) | | --- | | B.1.2 Implicit (please specify) | | B.1.3 Not stated/unclear (please specify) | | | --- | --- | --- | --- | --- | | B.2 Why was the study done at that point in time, in those contexts and with those people or institutions? *Please write in authors’ rationale if there is one. Elaborate if necessary, but indicate which aspects are reviewers’ interpretation.* | | B.2.1 Explicitly stated (please specify) | | --- | | B.2.2 Implicit (please specify) | | B.2.3 Not stated/unclear (please specify) | | | B.3 Was the study informed by, or linked to, an existing body of empirical and/or theoretical research? *Please write in authors’ description if there is one. Elaborate if necessary, but indicate which aspects are reviewers’ interpretation.* | | B.3.1 Explicitly stated (please specify) | | --- | | B.3.2 Implicit (please specify) | | B.3.3 Not stated/unclear (please specify) | | | B.4 Which of the following groups were consulted in working out the aims of the study, or issues to be addressed in the study? *Please write in authors’ description if there is one. Elaborate if necessary, but indicate which aspects are reviewers’ interpretation. Please cover details of how and why people were consulted and how they influenced the aims/issues to be addressed.* | | B.4.1 Researchers (please specify) | | --- | | B.4.2 Funder (please specify) | | B.4.3 Domestic Violence Specialist (please specify) | | B.4.4 Health Professionals (please specify) | | B.4.5 Allied Health(please specify) | | B.4.6 Non-health professionals (please specify) | | B.4.7 Women (please specify) | | B.4.8 Politicians (please specify) | | B.4.9 Government officials (please specify) | | B.4.10 Other education practitioner (please specify) | | B.4.11 Other (please specify) | | B.4.12 None/Not stated | | B.4.13 Coding is based on: Authors' description | | B.4.14 Coding is based on: Reviewers’ inference | | | B.5 Do authors report how the study was funded? | | B.5.1 Explicitly stated (please specify) | | --- | | B.5.2 Implicit (please specify) | | B.5.3 Not stated/unclear (please specify) | | | B.6 When was the study carried out? *If the authors give a year, or range of years, then put that in. If not, give a ‘not later than’ date by looking for a date of first submission to the journal, or for clues like the publication dates of other reports from the study.* | | B.6.1 Explicitly stated (please specify ) | | --- | | B.6.2 Implicit (please specify) | | B.6.3 Not stated/unclear (please specify) | | |

| **Section C: Study research question(s) and its policy or practice focus** *The first eight questions come from Keywording – please note if there is disagreement with previous coding*   | C.1 What is/are the topic focus/foci of the study? *Note this has already been asked in keyword Q6. Please use this question to provide further details* | | C.1.1 Abortion (please specify) | | --- | | C.1.2 Domestic Violence (please specify) | | C.1.3 Sexual Assault (please specify) | | C.1.4 Family Planning/Contraception (please specify) | | C.1.5 Screening (please specify) | | C.1.6 Forensic Nursing (please specify) | | C.1.7 Conscientious objectors (please specify) | | C.1.8 Sexual Coercion (please specify) | | C.1.9 Health professional Education (please specify) | | C.1.10 Other ( please specify) | | C.1.11 Coding is based on: Authors' description | | C.1.12 Coding is based on: Reviewers' inference | | | --- | --- | --- | --- | --- | --- | --- | --- | --- | --- | --- | --- | --- | --- | | C.2 What is the context of the study, if any? *Note this has already been asked in keyword Q6a. Please use this question to provide further detail if applicable.* | | C.2.1 General Practice | | --- | | C.2.2 Abortion Facility | | C.2.3 Family Planning Practice | | C.2.4 Midwifery Outreach | | C.2.5 Operating Rooms/Theatre | | C.2.6 Emergency Department | | C.2.7 Surgical Unit | | C.2.8 Specialist Telehealth/Outreach Clinic | | C.2.9 Rape Crisis Centre | | C.2.10 Coding is based on: Authors' description | | C.2.11 Coding is based on: Reviewers' inference | | | C.3 What is/are the population focus/foci of the study?  *Note this has already been asked in keyword Q8. Please use this question to provide further detail.* | | C.3.1 Women seeking abortions | | --- | | C.3.2 Victims of Rape | | C.3.3 Nurses | | C.3.4 Teachers as learners | | C.3.5 Non-teaching staff | | C.3.6 Other education practitioners | | C.3.7 Government | | C.3.8 Local education authority officers | | C.3.9 Parents | | C.3.10 Governors | | C.3.11 Other | | C.3.12 Coding is based on: Authors' description | | C.3.13 Coding is based on: Reviewers' inference | | | C.4 If learners are the population focus of the study, what is the relevant age group? *Note this has already been asked in keyword Q8a. Please use this question to provide further details.  This question is asking about the population focus i.e. the learners the study was aiming to make conclusions about. This may not necessarily be the same as the actual sample (or aimed for sample) of participants.  If this a study of teachers - tick 'not applicable'.* | | C.4.1 Not applicable (focus not learners) | | --- | | C.4.2 0-4 | | C.4.3 5-10 | | C.4.4 11-16 | | C.4.5 17-20 | | C.4.6 21 and over | | C.4.7 Coding is based on: Authors' description | | C.4.8 Coding is based on: Reviewers' inference | | | C.5 If learners are the population focus of the study, what is the sex of the learners? *Note this has already been asked in keyword Q8b. Please use this question to provide further details.  This question is asking about the population focus i.e. the learners the study was aiming to make conclusions about. This may not necessarily be the same as the actual sample (or aimed for sample) of participants.  If this a study of teachers - tick 'not applicable'.* | | C.5.1 Not applicable (focus not learners) | | --- | | C.5.2 Female only | | C.5.3 Male only | | C.5.4 Mixed sex | | C.5.5 Not stated/unclear | | C.5.6 Coding is based on: Authors' description | | C.5.7 Coding is based on: Reviewers' inference | | | C.6 What is/are the educational setting(s) of the study? *Note this has already been asked in keyword Q9. Please use this question to provide further details.* | | C.6.1 Community centre | | --- | | C.6.2 Correctional institution | | C.6.3 Government department | | C.6.4 Higher education institution | | C.6.5 General Practitioners | | C.6.6 Midwives | | C.6.7 Operating Room Staff | | C.6.8 Victims of DV | | C.6.9 Victims of sexual coercion | | C.6.10 Victims of incest | | C.6.11 Coding is based on: Authors' description | | C.6.12 Coding is based on: Reviewers' inference | | | C.7 In which country or countries was the study carried out? *Note this has already been asked in keyword Q5. Please use this question to provide further details, where relevant e.g. region or city.* | | C.7.1 Explicitly stated (please specify) | | --- | | C.7.2 Not stated/unclear (please specify) | | | C.8 If a programme or intervention is being studied, does it have a formal name?  *Note this has already been asked in keyword Q7. Please use this question to provide further details if applicable.* | | C.8.1 Not applicable (no programme or intervention) | | --- | | C.8.2 Yes (please specify) | | C.8.3 No (please specify) | | C.8.4 Not stated/unclear (please specify) | | | C.9 Please describe in more detail the specific phenomena, factors, services or interventions with which the study is concerned. *The questions so far have asked about the aims of the study and any named programme under study, but this may not fuly capture what the study is about. Please state or clarify here.* | | C.9.1 Details | | --- | | | C.10 What are the study research questions and/or hypotheses? *Research questions or hypotheses operationalise the aims of the study. Please write in authors’ description if there is one. Elaborate if necessary, but indicate which aspects are reviewers’ interpretation.* | | C.10.1 Explicitly stated (please specify) | | --- | | C.10.2 Implicit (please specify) | | C.10.3 Not stated/unclear (please specify) | | |
| --- | --- | --- | --- | --- | --- | --- | --- | --- | --- | --- | --- | --- | --- | --- | --- | --- | --- | --- | --- | --- | --- | --- | --- | --- | --- | --- | --- | --- | --- | --- | --- | --- | --- | --- | --- | --- | --- | --- | --- | --- | --- | --- | --- | --- | --- | --- | --- | --- | --- | --- | --- | --- | --- | --- | --- | --- | --- | --- | --- | --- | --- | --- | --- | --- | --- | --- | --- | --- | --- | --- | --- | --- | --- | --- | --- | --- | --- | --- | --- | --- | --- | --- | --- | --- | --- | --- | --- | --- | --- | --- | --- | --- | --- |

| **Section D: Methods - Design** *The EPPI Centre team and the Review Groups are working on more detailed questions that are appropriate to different types of studies. In the meantime, please add extra comments on the methods if you think they are relevant, or suggest questions.*   | D.1 Which type(s) of study does this report describe?  *Multiple codings accepted, but please take care to code in relation to the main focus of this data extraction.  Note this has already been asked in keyword Q10.   (Studies that look at the development of methodology, or that review primary research, are not addressed by data extraction.)* | | D.1.1 A. Description | | --- | | D.1.2 B. Exploration of relationships | | D.1.3 Ca. Evaluation: Naturally occurring | | D.1.4 Cb. Evaluation: Researcher-manipulated | | | --- | --- | --- | --- | --- | --- | | D.2 Which variables or concepts, if any, does the study aim to measure or examine? | | D.2.1 Explicitly stated (please specify) | | --- | | D.2.2 Implicit (please specify) | | D.2.3 Not stated/unclear (please specify) | | | D.3 Study timing  *Please indicate all that apply and give further details where possible.  · If the study examines one or more samples, but each at only one point in time it is cross-sectional.   · If the study examines the same samples, but as they have changed over time, it is retrospective, provided that the interest is in starting at one timepoint and looking backwards over time.  · If the study examines the same samples as they have changed over time and if data are collected forward over time, it is prospective provided that the interest is in starting at one timepoint and looking forward in time.* | | D.3.1 Cross-sectional | | --- | | D.3.2 Retrospective | | D.3.3 Prospective | | D.3.4 Not stated/unclear (please specify) | | | D.4 If the study is an evaluation, when were measurements of the variable(s) used for outcome made, in relation to the intervention? *If at least one of the outcome variables is measured both before and after the intervention, please use the before and after category.* | | D.4.1 Not applicable (not an evaluation) | | --- | | D.4.2 Before and after | | D.4.3 Only after | | D.4.4 Other (please specify) | | D.4.5 Not stated/unclear (please specify) | | |
| --- | --- | --- | --- | --- | --- | --- | --- | --- | --- | --- | --- | --- | --- | --- | --- | --- | --- | --- | --- | --- | --- | --- | --- | --- |

| **Section E: Methods - Groups**   | E.1 If comparisons are being made between two or more groups, please specify the basis of any divisions made for making these comparisons. *Please give further details where possible.* | | E.1.1 Not applicable (not more than one group) | | --- | | E.1.2 Prospective allocation into more than one group *(e.g. allocation to different interventions, or allocation to intervention and control groups)* | | E.1.3 No prospective allocation but use of pre-existing differences to create comparison groups  *(e.g. receiving different interventions, or characterised by different levels of a variable such as social class)* | | E.1.4 Other (please specify) | | E.1.5 Not stated/unclear (please specify) | | | --- | --- | --- | --- | --- | --- | --- | | E.2 How do the groups differ? | | E.2.1 Not applicable (not more than one group) | | --- | | E.2.2 Explicitly stated (please specify) | | E.2.3 Implicit (please specify) | | E.2.4 Not stated/unclear (please specify) | | | E.3 Number of groups *For instance, in studies in which comparisons are made between groups, this may be the number of groups into which the dataset is divided for analysis (e.g. social class, or form size), or the number of groups allocated to, or receiving, an intervention.* | | E.3.1 Not applicable (not more than one group) | | --- | | E.3.2 One | | E.3.3 Two | | E.3.4 Three | | E.3.5 Four or more (please specify) | | E.3.6 Other/unclear (please specify) | | | E.4 If prospective allocation into more than one group, what was the unit of allocation? *Please indicate all that apply and give further details where possible.* | | E.4.1 Not applicable (not more than one group) | | --- | | E.4.2 Not applicable (no prospective allocation) | | E.4.3 Individuals | | E.4.4 Groupings or clusters of individuals (details) *(e.g. classes of schools)* | | E.4.5 Other (e.g. individuals or groups acting as their own controls) (please specify) | | E.4.6 Not stated/unclear (please specify) | | | E.5 If prospective allocation into more than one group, which method was used to generate the allocation sequence? | | E.5.1 Not applicable (not more than one group) | | --- | | E.5.2 Not applicable (no prospective allocation) | | E.5.3 Random | | E.5.4 Quasi-random | | E.5.5 Non-random | | E.5.6 Not stated/unclear (please specify) | | | E.6 Where there was prospective allocation to more than one group, was the allocation sequence concealed from participants and those enrolling them until after enrolment? *Bias can be introduced, consciously or otherwise, if the allocation of pupils or classes or schools to a programme or intervention is made in the knowledge of key characteristics of those allocated. For example: children with more serious reading difficulty might be seen as in greater need and might be more likely to be allocated to the 'new' programme, or the opposite might happen. Either would introduce bias.* | | E.6.1 Not applicable (not more than one group) | | --- | | E.6.2 Not applicable (no prospective allocation) | | E.6.3 Yes (please specify) | | E.6.4 No (please specify) | | E.6.5 Not stated/unclear (please specify) | | | E.7 Study design summary  *In addition to answering the questions in this section, describe the study design in your own words. You may want to draw upon and elaborate the answers you have already given.* | | E.7.1 Details | | --- | | |
| --- | --- | --- | --- | --- | --- | --- | --- | --- | --- | --- | --- | --- | --- | --- | --- | --- | --- | --- | --- | --- | --- | --- | --- | --- | --- | --- | --- | --- | --- | --- | --- | --- | --- | --- | --- | --- | --- | --- | --- | --- | --- | --- | --- | --- | --- | --- | --- |

| **Section F: Methods - Sampling strategy**   | F.1 Are the authors trying to produce findings that are representative of a given population?  *Please write in authors’ description. If authors do not specify, please indicate reviewers’ interpretation.* | | F.1.1 Explicitly stated (please specify) | | --- | | F.1.2 Implicit (please specify) | | F.1.3 Not stated/unclear (please specify) | | | --- | --- | --- | --- | --- | | F.2 Which methods does the study use to identify people, or groups of people, to sample from and what is the sampling frame? *e.g. telephone directory, electoral register, postcode, school listing etc. There may be two stages – e.g.first sampling schools and then classes or pupils within them.* | | F.2.1 Not applicable (please specify) | | --- | | F.2.2 Explicitly stated (please specify) | | F.2.3 Implicit (please specify) | | F.2.4 Not stated/unclear (please specify) | | | F.3 Which methods does the study use to select people, or groups of people (from the sampling frame)? *e.g. selecting people at random, systematically - selecting for example every 5th person, purposively in order to reach a quota for a given characteristic.* | | F.3.1 Not applicable (no sampling frame) | | --- | | F.3.2 Explicitly stated (please specify) | | F.3.3 Implicit (please specify) | | F.3.4 Not stated/unclear (please specify) | | | F.4 Planned sample size *If more than one group, please give details for each group separately.* | | F.4.1 Not applicable (please specify) | | --- | | F.4.2 Explicitly stated (please specify) | | F.4.3 Not stated/unclear (please specify) | | |
| --- | --- | --- | --- | --- | --- | --- | --- | --- | --- | --- | --- | --- | --- | --- | --- | --- | --- | --- | --- | --- | --- | --- |
| **Section G: Methods - Recruitment and consent**   | G.1 Which methods are used to recruit people into the study? *e.g. letters of invitation, telephone contact, face-to-face contact.* | | G.1.1 Not applicable (please specify) | | --- | | G.1.2 Explicitly stated (please specify) | | G.1.3 Implicit (please specify) | | G.1.4 Not stated/unclear (please specify) | | | --- | --- | --- | --- | --- | --- | | G.2 Were any incentives provided to recruit people into the study? | | G.2.1 Not applicable (please specify) | | --- | | G.2.2 Explicitly stated (please specify) | | G.2.3 Not stated/unclear (please specify) | | | G.3 Was consent sought? *Please comment on the quality of consent if relevant* | | G.3.1 Not applicable (please specify) | | --- | | G.3.2 Participant consent sought | | G.3.3 Parental consent sought | | G.3.4 Other consent sought | | G.3.5 Consent not sought | | G.3.6 Not stated/unclear (please specify) | | | G.4 Are there any other details relevant to recruitment and consent? | | G.4.1 No | | --- | | G.4.2 Yes (please specify) | | |
| **Section H: Methods - Actual sample**   | H.1 What was the total number of participants in the study (the actual sample)? *If more than one group is being compared, please give numbers for each group.* | | H.1.1 Not applicable (e.g. study of policies, documents etc) | | --- | | H.1.2 Explicitly stated (please specify) | | H.1.3 Implicit (please specify) | | H.1.4 Not stated/unclear (please specify) | | | --- | --- | --- | --- | --- | --- | | H.2 What is the proportion of those selected for the study who actually participated in the study?  *Please specify numbers and percentages if possible.* | | H.2.1 Not applicable (e.g. study of policies, documents etc) | | --- | | H.2.2 Explicitly stated (please specify) | | H.2.3 Implicit (please specify) | | H.2.4 Not stated/unclear (please specify) | | | H.3 Which country/countries are the individuals in the actual sample from? *If UK, please distinguish between England, Scotland, N. Ireland and Wales, if possible. If from different countries, please give numbers for each.   If more than one group is being compared, please describe for each group.* | | H.3.1 Not applicable (e.g. study of policies, documents etc) | | --- | | H.3.2 Explicitly stated (please specify) | | H.3.3 Implicit (please specify) | | H.3.4 Not stated/unclear (please specify) | | | H.4 If the individuals in the actual sample are involved with a health service, which type of institution is it? *Please give details of the institutions, (e.g. size, geographic location, mixed/single sex etc) as described by the authors. If individuals are from different institutions, please give numbers for each. If more than one group is being compared, please describe all of the above for each group.* | | H.4.1 Not applicable (e.g. study of policies, documents etc) | | --- | | H.4.2 Community health (please specify) | | H.4.3 Correctional health (please specify) | | H.4.4 Government Hospital (please specify) | | H.4.5 Private Hopital (please specify) | | H.4.6 Private Clinic (please specify) | | H.4.7 Publically funded clinic(please specify) | | H.4.8 Private Telehealth (please specify) | | H.4.9 Public Telehealth (please specify) | | H.4.17 Other health setting (please specify) | | H.4.18 Coding is based on: Authors' description | | H.4.19 Coding is based on: Reviewers' inference | | | H.5 What ages are covered by the actual sample? *Please give the numbers of the sample that fall within each of the given categories. If necessary refer to a page number in the report (e.g. for a useful table).  If more than one group is being compared, please describe for each group.  If follow-up study, age at entry to the study.* | | H.5.1 Not applicable (e.g. study of policies, documents etc) | | --- | | H.5.2 0 to 4 | | H.5.3 5 to 10 | | H.5.4 11 to 16 | | H.5.5 17 to 20 | | H.5.6 21 and over | | H.5.7 Not stated/unclear (please specify) | | H.5.8 Coding is based on: Authors' description | | H.5.9 Coding is based on: Reviewers' inference | | | H.6 What is the sex of the individuals in the actual sample? *Please give the numbers of the sample that fall within each of the given categories. If necessary refer to a page number in the report (e.g. for a useful table).  If more than one group is being compared, please describe for each group.* | | H.6.1 Not applicable (e.g. study of policies, documents etc) | | --- | | H.6.2 Single sex (please specify) | | H.6.3 Mixed sex (please specify) | | H.6.4 Not stated/unclear (please specify) | | H.6.5 Coding is based on: Authors' description | | H.6.6 Coding is based on: Reviewers' inference | | | H.7 What is the socio-economic status of the individuals within the actual sample? *If more than one group is being compared, please describe for each group.* | | H.7.1 Not applicable (e.g. study of policies, documents etc) | | --- | | H.7.2 Explicitly stated (please specify) | | H.7.3 Implicit (please specify) | | H.7.4 Not stated/unclear (please specify) | | | H.8 What is the ethnicity of the individuals within the actual sample? *If more than one group is being compared, please describe for each group.* | | H.8.1 Not applicable (e.g. study of policies, documents etc) | | --- | | H.8.2 Explicitly stated (please specify) | | H.8.3 Implicit (please specify) | | H.8.4 Not stated/unclear (please specify) | | | H.9 What is known about the special educational needs of individuals within the actual sample? *e.g. specific learning, physical, emotional, behavioural, intellectual difficulties.* | | H.9.1 Not applicable (e.g. study of policies, documents etc) | | --- | | H.9.2 Explicitly stated (please specify) | | H.9.3 Implicit (please specify) | | H.9.4 Not stated/unclear (please specify) | | | H.10 Is there any other useful information about the study participants? | | H.10.1 Not applicable (e.g. study of policies, documents etc) | | --- | | H.10.2 Explicitly stated (please specify no/s.) | | H.10.3 Implicit (please specify) | | H.10.4 Not stated/unclear (please specify) | | | H.11 How representative was the achieved sample (as recruited at the start of the study) in relation to the aims of the sampling frame? *Please specify basis for your decision.* | | H.11.1 Not applicable (e.g. study of policies, documents etc) | | --- | | H.11.2 Not applicable (no sampling frame) | | H.11.3 High (please specify) | | H.11.4 Medium (please specify) | | H.11.5 Low (please specify) | | H.11.6 Unclear (please specify) | | | H.12 If the study involves studying samples prospectively over time, what proportion of the sample dropped out over the course of the study? *If the study involves more than one group, please give drop-out rates for each group separately. If necessary refer to a page number in the report (e.g. for a useful table).* | | H.12.1 Not applicable (e.g. study of policies, documents etc) | | --- | | H.12.2 Not applicable (not following samples prospectively over time) | | H.12.3 Explicitly stated (please specify) | | H.12.4 Implicit (please specify) | | H.12.5 Not stated/unclear | | | H.13 For studies that involve following samples prospectively over time, do the authors provide any information on whether and/or how those who dropped out of the study differ from those who remained in the study? | | H.13.1 Not applicable (e.g. study of policies, documents etc) | | --- | | H.13.2 Not applicable (not following samples prospectively over time) | | H.13.3 Not applicable (no drop outs) | | H.13.4 Yes (please specify) | | H.13.5 No | | | H.14 If the study involves following samples prospectively over time, do authors provide baseline values of key variables such as those being used as outcomes and relevant socio-demographic variables? | | H.14.1 Not applicable (e.g. study of policies, documents etc) | | --- | | H.14.2 Not applicable (not following samples prospectively over time) | | H.14.3 Yes (please specify) | | H.14.4 No | | |
| **Section I: Methods - Data collection**   | I.1 Please describe the main types of data collected and specify if they were used (a) to define the sample; (b) to measure aspects of the sample as findings of the study? *Only detail if more specific than Question D2* | | I.1.1 Details | | --- | | | --- | --- | --- | | I.2 Which methods were used to collect the data? *Please indicate all that apply and give further detail where possible.* | | I.2.1 Focus group | | --- | | I.2.2 Group interview | | I.2.3 One to one interview (face to face or by phone) | | I.2.4 Observation | | I.2.5 Self-completion questionnaire | | I.2.6 Self-completion report or diary | | I.2.7 Exams | | I.2.8 Clinical test | | I.2.9 Practical test | | I.2.10 Psychological test | | I.2.11 Hypothetical scenario including vignettes | | I.2.13 Health records | | I.2.14 Secondary data such as publicly available statistics | | I.2.15 Other documentation | | I.2.16 Not stated/unclear (please specify) | | I.2.17 Coding is based on: Authors' description | | I.2.18 Coding is based on: Reviewers' inference | | | I.3 Details of data collection methods or tool(s). *Please provide details including names for all tools used to collect data, and examples of any questions/items given. Also, please state whether source is cited in the report.* | | I.3.1 Explicitly stated (please specify) | | --- | | I.3.2 Implicit (please specify) | | I.3.3 Not stated/unclear (please specify) | | | I.4 Who collected the data? *Please indicate all that apply and give further detail where possible.* | | I.4.1 Researcher | | --- | | I.4.2 Doctor | | I.4.3 Nurse | | I.4.4 Other Health Professional | | I.4.5 Victims of DV | | I.4.6 Victims of Rape | | I.4.7 Victims of Incest | | I.4.8 Other hea practitioner | | I.4.9 Other (please specify) | | I.4.10 Not stated/unclear | | I.4.11 Coding is based on: Authors' description | | I.4.12 Coding is based on: Reviewers' inference | | | I.5 Do the authors describe any ways they addressed the reliability of their data collection tools/methods? *e.g. test - re-test methods  (Where more than one tool was employed, please provide details for each.)* | | I.5.1 Details | | --- | | | I.6 Do the authors describe any ways they have addressed the validity of their data collection tools/methods? *e.g. mention previous validation of tools, published version of tools, involvement of target population in development of tools.   (Where more than one tool was employed, please provide details for each.)* | | I.6.1 Details | | --- | | | I.7 Was there concealment of study allocation or other key factors from those carrying out measurement of outcome – if relevant?  *Not applicable – e.g. analysis of existing data, qualitative study. No – e.g. assessment of reading progress for dyslexic pupils done by teacher who provided intervention.*  *Yes – e.g. researcher assessing pupil knowledge of drugs - unaware of pupil allocation.* | | I.7.1 Not applicable (please say why) | | --- | | I.7.2 Yes (please specify) | | I.7.3 No (please specify) | | | I.8 Where were the data collected? *e.g. school, home.* | | I.8.1 Explicitly stated (please specify) | | --- | | I.8.2 Implicit (please specify) | | I.8.3 Unclear/not stated (please specify) | | | I.9 Any there other important features of data collection? *e.g. use of video or audio tape; ethical issues such as confidentiality etc.* | | I.9.1 Details | | --- | | |

| **Section J: Methods - Data analysis** *A section on statistical analysis is being developed for use where relevant. In the meantime please add any comments about statistical analysis.*   | J.1 Which methods were used to analyse the data? *Please give details eg. for in-depth interviews, how were the data handled? Details of statistical analysis can be given next.* | | J.1.1 Explicitly stated (please specify) | | --- | | J.1.2 Implicit (please specify) | | J.1.3 Not stated/unclear (please specify) | | | --- | --- | --- | --- | --- | | J.2 Which statistical methods, if any, were used in the analysis? | | J.2.1 Details | | --- | | | J.3 What rationale do the authors give for the methods of analysis for the study? *e.g. for their methods of sampling, data collection or analysis.* | | J.3.1 Details | | --- | | | J.4 For evaluation studies that use prospective allocation please specify the basis on which data analysis was carried out.  *'Intention to intervene' means that data were analysed on the basis of the original number of participants as recruited into the different groups.  'Intervention received' means data were analysed on the basis of the number of participants actually receiving the intervention.* | | J.4.1 Not applicable (not an evaluation study with prospective allocation) | | --- | | J.4.2 'Intention to intervene' | | J.4.3 'Intervention received' | | J.4.4 Not stated/unclear (please specify) | | | J.5 Do the authors describe any ways they have addressed the reliability of data analysis? *e.g. using more than one researcher to analyse data, looking for negative cases.* | | J.5.1 Details | | --- | | | J.6 Do the authors describe any ways they have addressed the validity of data analysis? *e.g. internal or external consistency; checking results with participants.* | | J.6.1 Details | | --- | | | J.7 Do the authors describe strategies used in the analysis to control for bias from confounding variables? | | J.7.1 Details | | --- | | | J.8 Please describe any other important features of the analysis. | | J.8.1 Details | | --- | | | J.9 Please comment on any other analytic or statistical issues, if relevant. | | J.9.1 Details | | --- | | |
| --- | --- | --- | --- | --- | --- | --- | --- | --- | --- | --- | --- | --- | --- | --- | --- | --- | --- | --- | --- | --- | --- | --- | --- | --- | --- | --- | --- | --- | --- | --- | --- | --- |
| **Section K: Results and Conclusions**   | K.1 How are the results of the study presented? *e.g. as quotations/figures within text, in tables, appendices.* | | K.1.1 Details | | --- | | | --- | --- | --- | | K.2 What are the results of the study as reported by authors? *Please give details and refer to page numbers in the report(s) of the study, where necessary (e.g. for key tables).* | | K.2.1 Details | | --- | | | K.3 Are there any obvious shortcomings in the reporting of the data? | | K.3.1 Yes (please specify) | | --- | | K.3.2 No | | | K.4 Do the authors report on all variables they aimed to study as specified in their aims/research questions? *This excludes variables just used to describe the sample.* | | K.4.1 Yes (please specify) | | --- | | K.4.2 No | | | K.5 Do the authors state where the full, original data are stored? | | K.5.1 Yes (please specify | | --- | | K.5.2 No | | | K.6 What do the author(s) conclude about the findings of the study?  *Please give details and refer to page numbers in the report of the study, where necessary.* | | K.6.1 Details | | --- | | |

| **Section L: Quality of the study - Reporting**   | L.1 Is the context of the study adequately described?  *Consider your answer to questions: Why was this study done at this point in time, in those contexts and with those people or institutions?(Section B, question2) Was the study informed by, or linked to an existing body of empirical and/or theoretical research?  (Section B, question 3)*  *Which of the following groups were consulted in working out the aims to be addressed in the study? (Section B, question 4)  Do the authors report how the study was funded?  (Section B, question 5)  When was the study carried out?  (Section B, question 6)* | | L.1.1 Yes (please specify) | | --- | | L.1.2 No (please specify) | | | --- | --- | --- | --- | | L.2 Are the aims of the study clearly reported? *Consider your answer to questions: What are the broad aims of the study? (Section B, question 1) What are the study research questions and/or hypotheses?  (Section C, question 10)* | | L.2.1 Yes (please specify) | | --- | | L.2.2 No (please specify) | | | L.3 Is there an adequate description of the sample used in the study and how the sample was identified and recruited? *Consider your answer to all questions in Methods on ‘Sampling Strategy’, ‘Recruitment and Consent’, and ‘Actual Sample’.* | | L.3.1 Yes (please specify) | | --- | | L.3.2 No (please specify) | | | L.4 Is there an adequate description of the methods used in the study to collect data? *Consider your answer to the following questions in Section I:  Which methods were used to collect the data? Details of data collection methods or toolsWho collected the data? Do the authors describe the setting where the data were collected? Are there other important features of the data collection procedures?* | | L.4.1 Yes (please specify) | | --- | | L.4.2 No (please specify) | | | L.5 Is there an adequate description of the methods of data analysis? *Consider your answer to the following questions in Section J:  Which methods were used to analyse the data?  What statistical methods if any, were used in the analysis? Who carried out the data analysis?* | | L.5.1 Yes (please specify) | | --- | | L.5.2 No (please specify) | | | L.6 Is the study replicable from this report? | | L.6.1 Yes (please specify) | | --- | | L.6.2 No (please specify) | | | L.7 Do the authors avoid selective reporting bias? (e.g. do they report on all variables they aimed to study as specified in their aims/research questions?) | | L.7.1 Yes (please specify) | | --- | | L.7.2 No (please specify) | | |
| --- | --- | --- | --- | --- | --- | --- | --- | --- | --- | --- | --- | --- | --- | --- | --- | --- | --- | --- | --- | --- | --- | --- | --- | --- | --- | --- | --- | --- |
| **Section M: Quality of the study - Methods and data**   | M.1 Are there ethical concerns about the way the study was done? *Consider consent, funding, privacy, etc.* | | M.1.1 Yes, some concerns (please specify) | | --- | | M.1.2 No concerns | | | --- | --- | --- | --- | | M.2 Were students and/or parents appropriately involved in the design or conduct of the study? | | M.2.1 Yes, a lot (please specify) | | --- | | M.2.2 Yes, a little (please specify) | | M.2.3 No (please specify) | | | M.3 Is there sufficient justification for why the study was done the way it was? | | M.3.1 Yes (please specify) | | --- | | M.3.2 No (please specify) | | | M.4 Was the choice of research design appropriate for addressing the research question(s) posed? | | M.4.1 Yes (please specify) | | --- | | M.4.2 No (please specify) | | | M.5 Have sufficient attempts been made to establish the reliability of data collection methods and tools? *Consider your answer to the following question in Section I:  Do the authors describe any ways they have addressed the reliability of their data collection tools/methods?* | | M.5.1 Yes, good (please specify) | | --- | | M.5.2 Yes, some attempt (please specify) | | M.5.3 No, none (please specify) | | | M.6 Have sufficient attempts been made to establish the validity of data collection tools and methods? *Consider your answer to the following question in Section I: Do the authors describe any ways they have addressed the validity of their data collection tools/methods?* | | M.6.1 Yes, good (please specify) | | --- | | M.6.2 Yes, some attempt (please specify) | | M.6.3 No, none (please specify) | | | M.7 Have sufficient attempts been made to establish the reliability of data analysis? *Consider your answer to the following question in Section J: Do the authors describe any ways they have addressed the reliability of data analysis?* | | M.7.1 Yes (please specify) | | --- | | M.7.2 No (please specify) | | | M.8 Have sufficient attempts been made to establish the validity of data analysis? *Consider your answer to the following question in Section J:  Do the authors describe any ways they have addressed the validity of data analysis?* | | M.8.1 Yes, good (please specify) | | --- | | M.8.2 Yes, some attempt (please specify) | | M.8.3 No, none (please specify) | | | M.9 To what extent are the research design and methods employed able to rule out any other sources of error/bias which would lead to alternative explanations for the findings of the study? *e.g. (1) In an evaluation, was the process by which participants were allocated to,or otherwise received the factor being evaluated, concealed and not predictable in advance? If not, were sufficient substitute procedures employed with adequate rigour to rule out any alternative explanations of the findings which arise as a result? e.g. (2) Was the attrition rate low and, if applicable, similar between different groups?* | | M.9.1 A lot (please specify) | | --- | | M.9.2 A little (please specify) | | M.9.3 Not at all (please specify) | | | M.10 How generalisable are the study results? | | M.10.1 Details | | --- | | | M.11 Weight of evidence - A: Taking account of all quality assessment issues, can the study findings be trusted in answering the study question(s)? *In some studies it is difficult to distinguish between the findings of the study and the conclusions. In those cases, please code the trustworthiness of this combined results/conclusion. ** Please remember to complete the weight of evidence questions B-D which are in your review specific data extraction guidelines. *** | | M.11.1 High trustworthiness (please specify) | | --- | | M.11.2 Medium trustworthiness (please specify) | | M.11.3 Low trustworthiness (please specify) | | | M.12 Have sufficient attempts been made to justify the conclusions drawn from the findings so that the conclusions are trustworthy? | | M.12.1 Not applicable (results and conclusions inseparable) | | --- | | M.12.2 High trustworthiness | | M.12.3 Medium trustworthiness | | M.12.4 Low trustworthiness | | | M.13 In light of the above, do the reviewers differ from the authors over the findings or conclusions of the study?  *Please state what any difference is.* | | M.13.1 Not applicable (no difference in conclusions) | | --- | | M.13.2 Yes (please specify) | | |
| **Section N: Reviewing record** *THIS SECTION PROVIDES A RECORD OF THE REVIEW OF THE STUDY.*   | N.1 Sections completed *Please indicate sections completed.* | | N.1.1 Administrative Details | | --- | | N.1.2 Study aims and rationale | | N.1.3 Study research question(s) and its policy or practice focus | | N.1.4 Methods | | N.1.5 Results and conclusions | | N.1.6 Quality of the study | | N.1.7 Reviewing record | | | --- | --- | --- | --- | --- | --- | --- | --- | --- | | N.2 Please use this space here to give any general feedback about these data extraction guidelines. | | N.2.1 Details | | --- | | | N.3 Please use this space to give any feedback on how these guidelines apply to your Review Group's field of interest. | | N.3.1 Details | | --- | | |

Bottom of Form
